# The miswired brain: making connections from neurodevelopment to psychopathology

**DOI:** 10.1186/1741-7007-9-23

**Published:** 2011-04-12

**Authors:** Kevin J Mitchell

**Affiliations:** 1 Smurfit Institute of Genetics and Institute of Neuroscience, Trinity College Dublin, Dublin 2, Ireland

## 

Developmental neurobiologists have made great progress in elucidating the molecular mechanisms underlying nervous system development. There has been less focus, however, on the consequences when these processes go wrong. As the evidence increases that mutations in neurodevelopmental genes are associated with major psychiatric disorders, defining these consequences assumes paramount importance in elucidating pathogenic mechanisms.

## The convergence of psychiatry and developmental neurobiology

Mutations in many different genes controlling cell migration, axon guidance and especially synaptogenesis are being found at an increasing rate in patients with schizophrenia, autism, epilepsy, mental retardation and other disorders [1]. These discoveries are prompting a paradigm shift regarding models of the genetic architecture of these disorders, which can be seen to be highly heterogeneous and primarily due to rare mutations in any of a large number of different loci [2]. They also indicate that these distinct clinical categories share overlapping etiologies and strongly implicate neurodevelopmental processes in a significant proportion of cases.

In this context, investigating the genetics of neurodevelopment in animals assumes greater importance. Many researchers have used genetic approaches in model organisms to dissect how neurodevelopmental processes work - to infer the normal function of a protein and to identify the cellular processes it is involved in. The application of these approaches in mice has revealed a wealth of information on how the brain gets wired. The converse question - how the brain can be miswired - has received less direct attention.

With the evidence of relevance to human disease, the phenotypes that arise in mice due to mutations in neurodevelopmental genes become of interest in themselves, not just as indicators of the normal function of the gene. It is important to ask: what happens to brain circuitry when a mutation affecting a process such as cell migration or synaptogenesis is mutated? The primary defects due to impairment of that protein are just the start of the story. How does miswiring of the circuit affect its function? What are the secondary consequences of altered activity in developing circuits? How does the developing brain react to such changes? How does the ultimate anatomical outcome affect brain functions, as indexed by physiology and behavior? Answering these questions will be a major challenge for the future and will require an integration of expertise from diverse fields and disciplinary traditions [3].

## Pathways and trajectories

One of the central questions emerging in the field is how mutations in so many different genes can yield similar clinical phenotypes. One possibility is that many of those genes' products collaborate in specific pathways [4,5]. Comparison of the primary defects that arise across various mutants will help to group them into biochemical pathways controlling specific cellular processes. Mutations in genes encoding proteins in the same pathways, may, in humans, be expected to give more similar clinical outcomes and possibly identify distinct subsets of patients [6].

An alternative, however, is that the phenotypic convergence emerges secondarily, in how the brain responds to what may be a wide range of primary defects. Homeostatic processes that normally function to maintain circuit parameters within an optimal range may, in the context of an initially miswired network, become maladaptive and channel the network into a pathological state [7]. The hypothesized emergence of psychosis due to maladaptive homeostasis in the dopaminergic pathway represents one possible example of such a phenomenon [8].

## Gene discovery

Evolutionary genetic arguments suggest there must be a very large number of disease susceptibility loci to explain the prevalence of common, deleterious heritable disorders [9]. The loci so far implicated in psychiatric disorders in human patients collectively explain only a fraction of all cases, suggesting that a large number of susceptibility genes remain to be found. An important implication of the convergence of psychiatric genetics on neurodevelopmental genes is that other genes involved in neurodevelopment in mice become prime candidates to be these "missing genes". If we can define the phenotypic spectra and trajectories of known psychiatric disease mutations in mice, then it stands to reason that any other loci where mutations cause similar phenotypes in mice might result in a psychiatric disorder if mutated in humans.

The size of the human population means that Murphy's law can be applied to genomics: any gene that can be mutated, will be - in fact, already has been [10]. Whole-genome sequencing approaches will provide catalogues of probably hundreds of mutations in each of us that deleteriously affect protein function [11]. Figuring out which of these is pathogenic in any given patient may be very difficult, especially if mutations at any single locus are responsible for only a very small fraction of cases of any disorder. Statistical approaches based purely on the distribution of such mutations in human populations or their segregation in families will likely need to be supplemented with information from other sources in determining the overall evidence that any given mutation is pathogenic. Phenotypic comparisons in mice could thus provide a strong prior probability for the causal involvement of a specific mutation identified in human patients.

## Genetic architecture

The phenotypic effects of any given mutation are likely to be modified, perhaps strongly, by genetic background. Understanding such epistatic interactions may be crucial in explaining the imperfect segregation of mutations with mental illness in humans [1]. Whether these interactions can profitably be explored in mice remains an open question. While genetic background effects are common for neurodevelopmental and especially behavioral phenotypes, there is no reason to think that the particular background effects in inbred lines of mice have direct relevance to human populations. On the other hand, the analysis of mice carrying mutations in several genes can be highly informative as to whether they function in similar pathways and how the system responds to multiple hits. Expanding genetic analyses of neurodevelopment to explore those kinds of epistatic interactions will therefore likely shed additional light on genetic complexities of these disorders in humans.

This is an exciting time, as a new field emerges at the overlap of developmental neurobiology and psychiatric genetics. The initial discoveries in both humans and animals provide crucial entry points to explore - using the full arsenal of molecular, cellular and systems neuroscience approaches - how mutations in neurodevelopmental genes impact on neural circuit structure and function, ultimately resulting in psychopathology.
